# Lying in Bed with the Head High

**Published:** 1854-12

**Authors:** 


### MISCELLANEOUS ITEMS.

Lying in bed with the Head high.—It is often a question among people who are unaquainted with the anatomy and physiology of man, whether lying with the head exalted, or even with the body, was most wholesome. Most, consulting their own ease on this point, argue in favor of that which they prefer. Now, although many delight in bolstering up their heads at night, and sleep soundly, without injury, yet we declare it to be a dangerous habit. The vessels through which the blood passes from the heart to the head, are always lessened in their cavities when the head is resting in bed higher than the body, therefore in all diseases attended with fever, the head should be pretty nearly on a level with the body; and people ought to accustom themselves to sleep thus to avoid danger.—*Medical Journal.*

